# The Impact of Revascularization Surgery on Headaches in Association with Cerebrovascular Reactivity in Patients with Moyamoya Angiopathy

**DOI:** 10.3390/brainsci14100967

**Published:** 2024-09-26

**Authors:** Francy D. Gallego Moyano, Helena C. Janssen, Lashmi Venkatraghavan, David J. Mikulis, Hugo Andrade Barazarte, Ivan Radovanovic, Eef J. Hendriks, Joanna D. Schaafsma

**Affiliations:** 1 Department of Medicine, Division of Neurology, University Health Network (UHN), 399 Bathurst St., Toronto, ON M5T 2S8, Canada; d.gallegomoyano@hotmail.com (F.D.G.M.); helena.janssen@uhn.ca (H.C.J.); 2 Department of Anesthesiology, University Health Network (UHN), Toronto, ON M5T 2S8, Canada; lashmi.venkatraghavan@uhn.ca; 3 Division of Neuroradiology, Joint Department of Medical Imaging, University Health Network (UHN), Toronto, ON M5T 2S8, Canada; david.mikulis@uhn.ca (D.J.M.); eef.hendriks@uhn.ca (E.J.H.); 4 Department of Surgery, Division of Neurosurgery, University Health Network (UHN), Toronto, ON M5T 2S8, Canada; hugo.andradebarazarte@uhn.ca (H.A.B.); ivan.radovanovic@uhn.ca (I.R.)

**Keywords:** moyamoya angiopathy, headaches, hemodynamic, cerebrovascular insufficiency, extracranial intracranial arterial bypass

## Abstract

Background/Objectives: Headaches in Moyamoya angiopathy are common but poorly understood. We aimed to investigate if headaches in Moyamoya angiopathy improve after revascularization surgery and whether this is associated with improvement in cerebrovascular reactivity on MRI (CVR-MRI). Methods: We included consecutive adult patients with Moyamoya angiopathy who had chart data on headaches, CVR-MRI, and underwent extracranial–intracranial bypass surgery between January 2010 and September 2022 at a tertiary neurovascular referral center. Clinical and CVR-MR imaging data of all patients were collected through systematic chart review, complemented by standard-of-care headache questionnaires from patients who were operated between 2018 and 2022. We evaluated headache features and explored the association between headaches and CVR before and after revascularization surgery. Results: Fifty-nine patients were included (mean age 47 ± 14 years, 43 females (73%)); among them, 41/59 (69%) reported headaches pre-surgery. Headache improved in 28/41 (68%) patients after revascularization surgery with a reduction in pain severity (median VAS-score from 5/10 to 2.5/10; *p* = 0.002), analgesic use (from 84% to 40%; *p* = 0.007), and sick leave (from 60% to 16%; *p* < 0.001). Improvement in headaches was associated with improvement in CVR (OR 5.3; 95% CI: 1.2–23.5) and sick leave reduction (OR 1.4; 95% CI: 1.6–121.4). Conclusions: Headaches in Moyamoya angiopathy are common and disabling. They may improve in most patients after revascularization surgery and seem to be associated with improvement in CVR, supporting the hypothesis of a potential vascular origin of the headaches.

## 1. Introduction

Moyamoya angiopathy is characterized by a progressive stenosis of the terminal portion of the internal carotid artery and its main branches with development of compensatory collaterals, so-called Moyamoya vessels [1,2]. Moyamoya angiopathy comprises both Moyamoya disease and Moyamoya syndrome, where the first is idiopathic and the latter occurs in association with another medical condition [2]. Moyamoya angiopathy can cause hemodynamic insufficiency resulting in ischemic stroke and intracranial hemorrhage caused by rupture of frail collaterals, both associated with a high rate of disability and even death [2,3]. Treatment in adults consists of medical management and in selected cases, revascularization surgery through a direct extracranial–intracranial arterial (EC–IC) bypass, with or without an indirect bypass, to prevent ischemic or hemorrhagic stroke [4,5]. To decide on revascularization surgery, an assessment of the hemodynamic status is part of the standard work-up [4,5]. An advanced technique to assess the hemodynamic reserve capacity is blood-oxygenation-level-dependent (BOLD)-MRI in response to carbon dioxide (CO_2_), a vasodilatory stimulus. This cerebrovascular reactivity MRI (CVR-MRI) is increasingly being offered to patients with chronic steno-occlusive disease [6,7].

In patients with Moyamoya angiopathy, the clinical focus is on stroke-like symptoms [4,5]. Headaches in Moyamoya patients are common, but limited data are available on their pathophysiology, characteristics, impact on quality of life, and response to revascularization surgery [8].

We aimed to investigate the presence and features of headaches in patients with Moyamoya angiopathy, their association with hemodynamic reserve capacity on CVR-MRI, and response to revascularization surgery to enhance recognition and improve understanding of this potentially disabling symptom.

## 2. Materials and Methods

**Figure 1:**
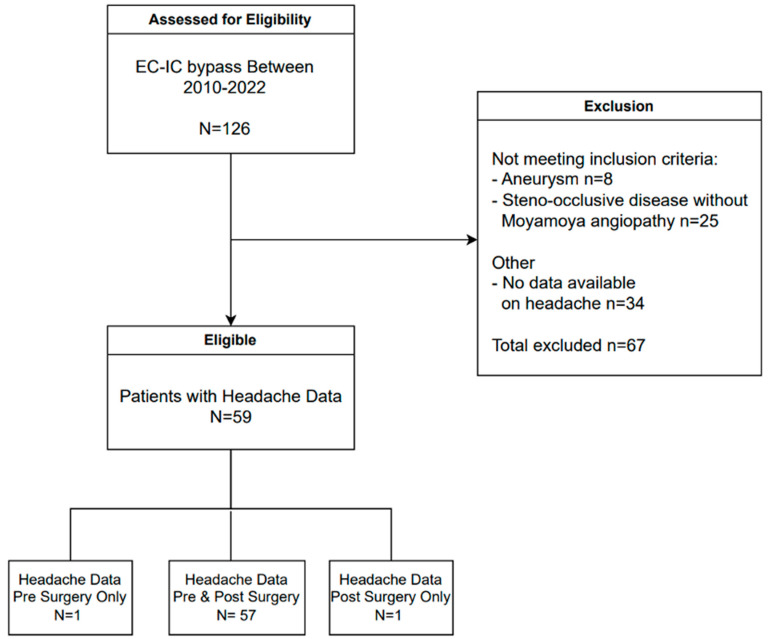
Patient selection. EC–IC bypass = extracranial–intracranial arterial bypass surgery.

From a prospective database of adult patients with cerebrovascular disease, who had at least one CVR-MRI study at our neurovascular tertiary referral center, we included consecutive patients with unilateral or bilateral Moyamoya angiopathy who underwent direct EC–IC bypass with or without indirect bypass surgery between January 2010 and September 2022 and had data on the presence or absence of headaches documented on their chart (Figure 1). We collected data on demographics, headache characteristics, CVR-MR imaging results, and type of flow augmentation surgery through systematic chart review.

CVR-MR imaging involved controlled end-tidal partial pressure of CO_2_ (P_ET_CO_2_) as a vasoactive stimulus delivered through a sealed mask (RespirAct, Thornhill Medical, Toronto, ON, Canada), and BOLD MRI at a 3.0-Tesla system. Full brain BOLD MR images were acquired using a T2*-weighted two-dimensional gradient-echo sequence with standard echo-planar readout. Three-dimensional T1-weighted anatomical MR images were acquired for co-registration with BOLD MR images using an inversion-recovery fast spoiled gradient-echo sequence. The calculated CVR was based on the BOLD-MR signal intensity related to the P_ET_CO_2_. This technique has been described previously in further detail [6,7]. A clinic visit followed CVR-MR imaging.

Patients who had surgery between 2018 and 2022 also completed a structured headache questionnaire as part of standard clinical practice. This questionnaire comprises the presence, localization, and types of headaches, headache triggers, accompanying symptoms, as well as analgesic use and the impact of headaches on mood and sick leave before and after surgery. It includes a Visual Analog Scale (VAS) score on a scale from 0 (no pain) to 10 (maximal pain).

The Institutional Research Ethics Board approved the study before data collection. The need for informed consent was waived.

### Statistical Analysis

In addition to descriptive analyses, we used the McNemar–Bowker test to compare proportions and the Wilcoxon signed rank test to compare VAS scores before and after surgery. A *p*-value < 0.05 was considered statistically significant. We explored the association between headache improvement and improvement of cerebrovascular reactivity after surgery using odds ratios with corresponding 95% confidence intervals (95% CI). Subsequently, a logistic regression analysis was employed to identify potential confounders for this association and examine other factors related to headache improvement after surgery. The data were statistically analyzed using SPSS version 28 software.

## 3. Results

Fifty-nine patients met the inclusion criteria (mean age 47 ± 14 years, 73% females) (Figure 1). Most patients (70%) were diagnosed with Moyamoya disease and 30% with Moyamoya syndrome. Thirty-eight patients (64%) were diagnosed with bilateral Moyamoya angiopathy, and 19 (50%) of them underwent bilateral flow augmentation surgery (Table 1).

### 3.1. Headaches before and after Flow Augmentation Surgery

Data on headaches both before and after surgery were available for 57/59 (97%) patients (Figure 1). Most patients (72%) reported headaches before bypass surgery, which improved in more than two-thirds (68%) of patients after surgery. Two patients developed new headaches after surgery (Table 2). The lateralization of the headache was not correlated with the side of Moyamoya arteriopathy (*p* = 0.317) nor with the side of surgery (*p* = 0.754).

Thirty-one patients who underwent flow augmentation surgery between 2018 and 2022 completed the headache questionnaire. Twenty-five (25/31; 81%) reported mostly throbbing or pressure-type headaches before surgery with a median headache severity of 5/10 on the VAS. Among these patients, 17/25 (68%) reported one or more headache triggers, and 15/25 (60%) described one or more accompanying symptoms, predominantly nausea/vomiting and a preceding aura. Most patients (84%) used one or more classes of analgesics. Almost half of the patients felt depressed due to their headaches, and nearly two-thirds of patients missed work or quit their jobs because of headaches (Table 3).

After surgery, sick leave decreased significantly, from 60% (15/25) to 16% (4/25) (*p* < 0.001), which was associated with headache improvement (OR 1.4; 95% CI: 1.6–121.4). Headache severity improved with a reduction in VAS score from 5/10 to 2.5/10 after surgery (*p* = 0.002), resulting in a reduction in analgesic use from 84% to 40% (*p* = 0.007) after surgery (Table 3).

### 3.2. Cerebrovascular Reserve before and after Flow Augmentation Surgery

Pre-operative CVR-MRI results were available for 57/59 patients (97%). Most patients (39/57 (68%)) had bilateral decreased CVR. Post-operative CVR-MRI results were available for 53/59 patients (90%). CVR improved in 36/53 patients (68%) and worsened in 6/53 patients (11%) after surgery. Improvement of CVR was associated with an improvement in headaches (OR 5.3; 95%CI: 1.2–23.5).

## 4. Discussion

In this cohort of patients with Moyamoya angiopathy, headaches were common, disabling, and associated with a high rate of sick leave. Following flow augmentation surgery, most patients experienced a reduction in headaches, along with observed improvement in cerebrovascular reserve on MRI. This led to a decreased use of analgesics and a reduction in sick leave.

Headaches in Moyamoya angiopathy are common but often remain unaddressed in clinical practice due to insufficient awareness of their impact on quality of life and a limited understanding of the underlying pathophysiology [8,9].

In a prior study including 55 patients with Moyamoya angiopathy, of whom 34 had flow augmentation surgery, a similar headache frequency before surgery was described [8]. Headaches improved after surgery in around two-thirds of the patients, consistent with our findings. Additionally, our analysis showed an association between headache reduction and improved cerebrovascular reserve on MRI after surgery, potentially offering insights into its underlying pathophysiological mechanism. When the cerebrovascular reserve capacity is compromised due to Moyamoya angiopathy, leptomeningeal collateral arteries typically dilate to compensate for reduced flow, which may cause headaches by stimulation of dural nociceptors [8,10,11]. The vasoactive hypothesis in migraine supports this theory and would explain the migraine-like headaches in Moyamoya angiopathy patients [12,13]. Another explanation for headaches in this patient population could be hypoxia and microvascular ischemia induced by chronic cerebral hypoperfusion and impaired hemodynamics from progressive intracranial steno-occlusive disease. Microvascular ischemia could generate cortical spreading depression, as is also seen in migraine with aura [8,10,14]. To support this hypothesis, future studies could focus on headaches and chronic ischemic changes on MRI in patients with Moyamoya angiopathy. 

Another potential explanation for headaches in this patient population could be concomitant depression. Psychological well-being, including depression, can improve after revascularization surgery in Moyamoya disease patients [15]. Feelings of depression improved after revascularization surgery in our cohort, though not significantly, and they were not associated with headache improvement, potentially because of lack of power.

Not only in adults, but also in pediatric patients with Moyamoya disease, headaches are common and respond to indirect revascularization surgery [9,16,17].

Interestingly, contrasting findings were reported by a Chinese observational study, which showed no differences in long-term outcomes of headaches between Moyamoya angiopathy patients who underwent revascularization surgery and those with conservative treatment [13]. It could be argued that patients receiving conservative management had less advanced disease compared to patients who required revascularization surgery and therefore had fewer headaches, potentially masking a positive effect of surgery on headaches.

As demonstrated in this study, the impact of headaches on the daily routine of patients with Moyamoya angiopathy is significant, which warrants increased awareness in clinical practice. In children with headaches caused by Moyamoya disease, a decrease in school attendance was seen [16]. The effect of revascularization surgery on headache severity and reduction in sick leave is significant and, therefore, patients and their families should be informed about these benefits when discussing surgery.

A strength of this study is the relatively large cohort size of Moyamoya angiopathy patients and the available standard-of-care headache questionnaire, both providing robust data on their clinical and radiographic status before and after revascularization surgery.

A limitation of this study is its retrospective nature and the potential introduction of reporting bias concerning the presence of headaches and recall bias regarding headache characteristics and changes. This limits the ability to establish causality and warrants careful drawing of conclusions. 

These study results need to be validated prospectively in a larger cohort of patients with Moyamoya angiopathy to mitigate recall and reporting bias. Once the pathophysiology of these disabling headaches is better understood, specific medical treatment targets may be identified to tailor headache management in this patient population.

## 5. Conclusions

Headaches in Moyamoya angiopathy were common and disabling, although they often improved after revascularization surgery, resulting in a better quality of life. Their association with improved cerebrovascular reactivity post-surgery supports the postulation that headaches in patients with Moyamoya angiopathy may have a vascular origin.

## Figures and Tables

**Table 1:** Baseline characteristics.

	n = 59
Age (yr)	47 ± 14
Female sex	43/59 (73%)
Moyamoya disease	41/59 (70%)
Unilateral	11/41 (27%)
Bilateral	30/41 (73%)
Moyamoya syndrome	18/59 (31%)
Unilateral	10/18 (56%)
Bilateral	8/18 (44%)
History of stroke	
Only ischemic	35/59 (59%)
Only hemorrhagic	6/59 (10%)
Ischemic and hemorrhagic	9/59 (15%)
No history of stroke	9/59 (15%)
Bypass surgery n (%)	
EC–IC bypass left	22/59 (37%)
EC–IC bypass right	18/59 (31%)
EC–IC bypass bilateral	19/59 (32%)
EC–IC bypass + EDAS	4/59 (7%)
Interval CVR-MRI & bypass surgery (median (IQR), months)	5 (3–8)
Interval bypass surgery & CVR-MRI (median (IQR), months)	6 (3–12)

EC–IC bypass: extracranial–intracranial arterial bypass surgery (direct); EDAS: encephaloduroarteriosynangiosis (indirect). IQR: interquartile range.

**Table 2:** Headaches before and after flow augmentation surgery.

	n (%)
Headaches before surgery	41/57 (72%)
Headaches improved after surgery	28/41 (68%)
Headaches were unchanged after surgery	11/41 (27%)
Headaches worsened after surgery	2/41 (5%)
New onset headaches after surgery	2/57 (4%)

**Table 3:** Headache characteristics before and after flow augmentation surgery.

	Before Surgery n (%)	After Surgery n (%)
Completed questionnaires	31/59 (53%)	31/59 (53%)
Presence of headaches	25/31 (81%)	19/31 (61%)
Types of Headaches		
Throbbing headache	9/25 (36%)	6/19 (32%)
Pressure-type headache	7/25 (28%)	6/19 (32%)
Dull headache	6/25 (24%)	6/19 (32%)
Sharp headache	4/25 (16%)	2/19 (11%)
Localization of Headaches		
Bilateral	19/25 (76%)	14/19 (74%)
Unilateral	6/25 (24%)	5/19 (26%)
Headache Triggers		
Lack of sleep	6/25 (24%)	5/19 (26%)
Menses	4/25 (16%)	4/19 (21%)
Exercise	3/25 (12%)	1/19 (5%)
Mental stress	3/25 (12%)	2/19 (11%)
Weather changes	1/25 (4%)	1/19 (5%)
Accompanying Symptoms		
Preceding aura	6/25 (24%)	6/19 (32%)
Nausea/vomiting	9/25 (36%)	9/19 (47%)
Photo-/phonophobia	4/25 (16%)	3/19 (16%)
Dizziness	4/25 (16%)	2/19 (11%)
Transient focal weakness/sensory symptoms	2/25 (8%)	0/19 (0%)
Analgesic Use	21/25 (84%)	10/25 (40%) *
Acetaminophen	16/25 (64%)	5/25 (20%)
Non-steroidal anti-inflammatory drugs	5/25 (20%)	2/25 (8%)
Triptans	3/25 (12%)	1/25 (4%)
Anticonvulsants	5/25 (20%)	2/25 (8%)
Opioids (codeine, caffeine, morphine)	3/25 (12%)	3/25 (12%)
Multiple drug classes	7/25 (28%)	3/25 (12%)
Headache impact		
Pain severity: VAS-score (median (IQR))	5 (0–9)	2.5 (0–5) *
Feelings of depression	11/25 (44%)	8/25 (32%)
Sick leave/quit job	15/25 (60%)	4/25 (16%) *

VAS: Visual Analog Scale (0–10). IQR: interquartile range. * Statistically significant difference.

## Data Availability

The data that support the findings of this study are available from the corresponding author, J.D.S., upon reasonable request. The data are not publicly available due to privacy and ethical restrictions.
